# Quantitative tools for addressing hospital readmissions

**DOI:** 10.1186/1756-0500-5-620

**Published:** 2012-11-02

**Authors:** Ronald J Lagoe, Diane S Nanno, Mary E Luziani

**Affiliations:** 1 Hospital Executive Council, PO Box 35089, Syracuse, NY, 13235, USA; 2 Crouse Hospital, Syracuse, NY, 13210, USA; 3 St. Joseph’s Hospital Health Center, Syracuse, NY, 13203, USA

**Keywords:** Hospitalization, Quality assurance, Hospital readmissions

## Abstract

**Background:**

Increased interest in health care cost containment is focusing attention on reduction of hospital readmissions. Major payors have already developed financial penalties for providers that generate excess readmissions. This subject has benefitted from the development of resources such as the Potentially Preventable Readmissions software. This process has encouraged hospitals to renew efforts to improve these outcomes. The aim of this study was to describe quantitative tools such as definitions, risk estimation, and tracking of patients for reducing hospital readmissions.

**Findings:**

This study employed the Potentially Preventable Readmissions software to develop quantitative tools for addressing hospital readmissions. These tools included two definitions of readmissions that support identification and management of patients. They also included analytical approaches for estimation of the risk of readmission for individual patients by age, discharge status of the initial admission, and severity of illness. They also included patient specific spreadsheets for tracking of target populations and for evaluation of the impact of interventions.

**Conclusions:**

The study demonstrated that quantitative tools including the development of definitions of readmissions, estimation of the risk of readmission, and patient specific spreadsheets could contribute to the improvement of patient outcomes in hospitals.

## Findings

### Introduction

Available information suggests that, in the United States, the urgency of cost containment is increasing. Although the rise in health care expenses has mitigated somewhat in recent years, it is the major driver of recent increases in the cost of living [1-3].

This situation has generated renewed interest in linking health care costs containment with improvement of outcomes, such as reduction of hospital readmissions and inpatient complications [4]. Both Medicare and Medicaid have already developed financial penalties for hospital readmissions beyond certain criteria [5].

Historically, hospital readmissions have been evaluated and addressed by individual health care providers. It has been suggested that readmissions are more useful than variables such as inpatient mortality, because they are the only inpatient indicator that reflects the condition of the patient after hospital discharge [6,7].

During the 1990s, the availability of hospital discharge abstract data made it possible to develop consistent criteria for hospital readmissions and apply them to large populations of patients [8-10]. In the twenty first century, the development of computerized algorithms addressing hospital readmissions further increased the potential for evaluation and clinical management of these outcomes [11]. The use of these resources to address hospital readmissions has required health care providers to develop additional tools and mechanisms for clinical management.

One of the most fundamental mechanisms underlying hospital readmissions is their definitions, which vary. It has been suggested that readmissions may be defined as the number of patients who experience unplanned readmission within 30 days of the initial admission [12].

Another tool related to hospital readmissions that has been widely discussed is the evaluation of risk. Because acute hospitals admit large numbers of patients, it has been assumed that all of them cannot be followed for reduction of readmissions by clinical staff such as nurses and that models are needed to identify those at greatest risk of readmission. It has been suggested that the best models should have good predictive ability, be usable in large populations, use data that are easily available, and be based on indicators commonly used [13]. A number of predictive models of readmissions have been developed [14-16]. Some of these are based on administrative data, others require additional data collection. Many researchers have concluded that most models for prediction of readmissions are of poor quality [13,17,18].

The reduction of hospital readmissions by providers also requires criteria for tracking patients and specific interventions to prevent rehospitalizations. Tracking criteria are essential to ensure that target populations are being followed [19,20]. Effective interventions are a key to preventing these adverse outcomes. The avoidance of readmissions in patients with chronic diseases is a major challenge for these services, but worth the resources because of the impact of chronic diseases on health care costs [4,21-24].

This study described the development of quantitative tools related to management of hospital inpatient readmissions in a small metropolitan area of the United States, Syracuse, New York. Included in this aim were the development of a) Definitions of Inpatient Readmissions, b) Criteria for Evaluating and Tracking Target Populations, and c) Criteria for Evaluating the Risk of Readmission.

### Population and methods

The setting for the study, the service area of the Syracuse hospitals, includes an estimated population of 600,000 (New York Statistical Information System, 2010). The hospitals, with total inpatient discharges excluding well newborns for 2010 include St. Joseph’s Hospital Health Center (22,421), Crouse Hospital (20,448), University Hospital of the State University of New York Upstate Medical University (19,871), and Community General Hospital (7,369) (Hospital Executive Council, Unpublished data, 2011).

Historically, the Syracuse hospitals have worked cooperatively to improve the outcomes and efficiency of care in the community. A number of these efforts have been coordinated through the Hospital Executive Council, the collaborative planning organization for these facilities [25].

During 2010 and 2011, the Syracuse hospitals with the largest numbers of inpatient discharges, St. Joseph’s Hospital Health Center and Crouse Hospital, developed programs addressing reduction of inpatient readmissions in order to improve patient care and make best use of clinical resources. Through these efforts, the hospitals addressed a number of important technical issues and developed management tools related to the evaluation and tracking of inpatient readmissions.

These programs were supported by the Potentially Preventable Readmissions software produced by 3M™ Health Information Services. This resource uses hospital discharge abstract data to identify and track readmissions using standardized criteria [11].

### Definitions of inpatient readmissions

The definition of inpatient readmissions is of fundamental importance because it relates to the management of patients with respect to the indicators. The literature suggests that there is general agreement on a definition based on patients who return for repeat hospitalization within 30 days for non elective reasons [12].

Within these criteria, a definition of a hospital readmission based on a chain, made up of an initial admission and readmissions, emphasizes the initial admission as the driver. In this definition, the diagnosis of the initial admission becomes the diagnosis of the chain. A different definition of a hospital readmission, based on the individual readmissions, emphasizes these individual admissions. In this definition, the diagnoses of the individual readmissions are counted.

In the efforts of the Syracuse hospitals to develop programs for management of readmissions, both definitions were evaluated. This study provided examples and the respective implications of each for the patient management based on the Potentially Preventable Readmissions algorithm.

### Criteria for evaluating the risk of readmission

The development of criteria for predicting the risk of hospital readmissions was an essential component of efforts to address these adverse outcomes in the Syracuse hospitals.

The acute care facilities involved in the study each provided treatment for more than 13,000 adult medical and surgery inpatients during 2010. As not for profit general hospitals in a small metropolitan area, these acute care facilities had limited numbers of staff to follow patients within this population who might be readmitted. Based on this information, they developed analyses of the risk of readmissions for chronic diseases using indicators such as patient age, severity of illness, and discharge status. The objectives of these efforts focused on identification of indicators that accounted for the largest numbers of repeat hospitalizations according to the Potentially Preventable Readmissions algorithm.

### Criteria for identifying and tracking target populations

Efforts to address inpatient readmissions in the Syracuse hospitals also involved the development of spreadsheets for staff dedicated to tracking at risk patients. The tracking function was important because it would become the basis for interventions.

The development of interventions to address readmissions in the Syracuse hospitals was based on a planning process that involved identification of patients with chronic diseases who were readmitted to assess reasons for their rehospitalizations. Based on this process, spreadsheets for clinical management of patients were developed.

 The patient specific data used in this portion of the study were administrative data, rather than information derived directly from patient medical records. This information is obtained from hospital discharge abstracts, rather than patient medical records.Â The use of administrative data among the hospitals of Syracuse, New York is authorized through the Hospital Executive Council Review Committee. Such research was carried out in compliance with the Helsinki Declaration.

## Results

The study data focused on development of quantitative tools for managing hospital readmissions. These tools were developed by the staffs of Crouse Hospital, St. Joseph’s Hospital Health Center, and the Hospital Executive Council as part of efforts to reduce readmissions.

### Definitions of inpatient readmissions

The initial component of these efforts involved definitions of hospital readmissions.

Definitions were assembled within the parameters identified in the literature, including patients discharged from hospitals and returning as inpatients within 30 days for non elective reasons.

A readmission chain was defined as an initial inpatient admission followed by at least one readmission within the criteria. In this definition, the term followed referred to the readmission occurring after the initial admission in time, rather than some type of monitoring process by the hospital. Each chain was defined and designated by the initial admission. The number of chains was based on the number of initial admissions, regardless of the number of rehospitalizations. The diagnosis of the initial admission was defined as the diagnosis of the chain, regardless of the diagnoses of rehospitalizations that followed.

The second definition was based on individual readmissions within the criteria. The readmissions were defined and designated by the individual rehospitalizations. Initial admissions were followed by different numbers of readmissions. Again, the term followed referred to the readmission coming after the initial admission in time. The readmissions were designated by their own principal diagnoses, rather than the principal diagnoses of the chains.

In the Syracuse hospitals, each of these definitions was associated with different advantages for clinical management. The chain definition was based on principal diagnoses that could readily be identified and tracked before readmissions occurred. Likely candidates for readmission could be followed through patients with typical initial admission diagnoses, such as congestive heart failure and pneumonia. The definition based on individual readmissions focused on the diagnoses and the numbers of the individual rehospitalizations.

The readmission programs in the Syracuse hospitals included tracking of patients using both definitions. A sample of these data follows in Table 1.

**Table 1:** Potentially preventable readmissions within 30 days congestive heart failure - APR DRG 194 Crouse Hospital 2010 - 2011

	**Number of PPR chains**	**CHF discharges**	**Readmission rate**	**Number of readmissions**	**CHF discharges**	**Readmission rate**
2010						
January	7	38	18.4	7	38	18.4
February	5	38	13.2	21	38	55.3
March	4	25	16.0	18	25	72.0
Jan - Mar	16	101	15.8	46	101	45.5
April	7	37	18.9	20	37	54.1
May	4	32	12.5	14	32	43.8
June	5	31	16.1	13	31	41.9
Apr - Jun	16	100	16.0	47	100	47.0
July	6	29	20.7	13	29	44.8
August	7	33	21.2	12	33	36.4
September	1	39	2.6	27	39	69.2
Jul - Sept	14	101	13.9	52	101	51.5
October	4	27	14.8	17	27	63.0
November	4	40	10.0	26	40	65.0
December	2	27	7.4	9	27	33.3
Oct - Dec	10	94	10.6	52	94	55.3
Total 2010	56	396	14.1	197	396	49.7
2011						
January	5	32	15.6	11	32	34.4
February	1	28	3.6	7	28	25.0
March	2	38	5.3	6	38	15.8
Jan - Mar	8	98	8.2	24	98	24.5
April	2	33	6.1	9	33	27.3
May	5	35	14.3	10	35	28.6
June	7	29	24.1	15	29	51.7
Apr - Jun	14	97	14.4	34	97	35.1
July	5	42	11.9	11	42	26.2
August	0	26	0.0	7	26	26.9
September	3	28	10.7	7	28	25.0
Jul - Sept	8	96	8.3	25	96	26.0

This information, for congestive heart failure readmissions to Crouse Hospital, identifies different numbers of readmissions and rates based on the two definitions. Because the chain definition was based on numbers of initial admissions, frequencies in this category were 19 – 41 percent of those for the definition based on numbers of readmissions. Because the numbers of discharges in the denominators were the same, the rates for the two definitions varied by the same proportions.

### Criteria for evaluating the risk of readmission

As previously noted, the literature has suggested that a precise quantitative definition of the risk of hospital readmission has not yet been developed. In order to focus clinical management on patients with the highest risk of readmission, the Syracuse hospitals have identified readmission rates for chronic diagnoses based on indicators generally associated with readmissions such as age, discharge status, and severity of illness. Examples of these data are summarized in Table 2.

**Table 2:** Inpatient potentially preventable readmissions within 30 days congestive heart failure patients - APR DRG 194

**St. Joseph's Hospital Health Center July 2010 - June 2011**	**Readmission chains**	**At risk discharges**	**Readmission rate**
Age Level			
18 - 45 Years	1	7	14.3
45 - 64 Years	12	89	13.5
65 - 74 Years	10	108	9.3
75 - 84 Years	31	220	14.1
85 Years and Over	30	188	16.0
Total	83	612	13.6
Discharge Status			
Self Care	34	258	13.2
Home Care	25	235	10.6
Nursing Home	24	118	20.3
Deaths/Transfers	0	1	0.0
Total	83	612	13.6
Severity of Illness			
1 - Minor	3	21	14.3
2 - Moderate	35	264	13.3
3 - Major	39	280	13.9
4 - Extreme	6	47	12.8
Total	83	612	13.6
**Crouse Hospital January - June 2011**	**Readmission chains**	**At risk discharges**	**Readmission rate**
Age Level			
18 - 45 Years	0	2	0.0
45 - 64 Years	0	20	0.0
65 - 74 Years	0	46	0.0
75 - 84 Years	8	56	14.3
85 Years and Over	8	72	11.1
Total	16	196	8.2
Discharge Status			
Self Care	2	54	3.7
Home Care	8	84	9.5
Nursing Home	6	56	10.7
Deaths/Transfers	0	2	0.0
Total	16	196	8.2
Severity of Illness			
1 - Minor	0	10	0.0
2 - Moderate	2	68	2.9
3 - Major	12	100	12.0
4 - Extreme	2	18	11.1
Total	16	196	8.2

PPR Chains include numbers of patients with at least one return within 30 days, for non elective reasons, clinically related to the initial admission according to the 3M Potentially Preventable Readmissions software.

Sources: St. Joseph's Hospital Health Center; Crouse Hospital.

This information was based on the chain definition of readmissions with an initial admission diagnosis of congestive heart failure during at St. Joseph’s Hospital Health Center and Crouse Hospital. The breakdowns of numbers and rates of readmission within each indicator were employed in the planning of initiatives to identify the best opportunities for patient management. These breakdowns related to numbers and rates of readmission for each.

Within Table 2, the term “At Risk Discharges” generally refers to all discharges of patients with the readmission diagnosis being evaluated, in this case, congestive heart failure. This definition is based on criteria within the Potentially Preventable Readmissions software. For some patients, it has been adjusted to exclude diagnoses that were determined not to be directly related to the principal readmission diagnosis, such as secondary malignancies for patients with circulatory principal diagnoses.

The distributions of patients by age demonstrated that 73 percent of chain readmission population at St. Joseph’s Hospital Health Center and 100 percent of the chain readmission population at Crouse Hospital were aged 75 years or more. This demonstrated that although readmission rates for the age levels identified were similar, a substantial majority of these patients were frail elderly. Small percentages of the chain readmissions involved patients aged less than 75 years.

The distribution of patients by discharge status was based on the initial admission in each chain in order to evaluate the potential for readmission. For this indicator, the largest number of patients, 41.0 percent, were associated with initial discharges to self care, while the highest readmission rate was produced by initial discharges to nursing homes at St. Joseph’s Hospital Health Center. At Crouse Hospital, 50.0 percent were associated with discharges to home care, while the highest readmission rate was produced by initial discharges to nursing homes.

The distribution of patients by severity of illness indicated that the highest volumes were produced by patients at Major (47.0 percent) and Moderate (42.2 percent) severity of illness at St. Joseph’s Hospital Health Center, and Major (75.0 percent) severity of illness at Crouse Hospital. Readmission rates among most severity of illness categories were similar. Patients at Minor or Moderate severity of illness may have offered more potential for avoiding readmissions, however, these patients accounted for the minority of the study populations.

### Criteria for identifying and tracking target populations

Efforts to reduce hospital readmissions in Syracuse also involved the development of quantitative tools for tracking target populations and outcomes. Both of these functions were addressed through the development of patient specific spreadsheets based on Potentially Preventable Readmissions. The spreadsheets were used to identify and quantify the patient populations that should have been tracked and the chain readmissions in each chain month.

An example of these spreadsheets, including patient specific data for chain month September 2011 at St. Joseph’s Hospital Health Center, is summarized below.

The spreadsheet format contains retrospective data for use in evaluating target populations tracked. It includes readmission chains identified by initial discharge (IA) and readmission discharge (RA), with a specific principal diagnosis and All Patients Refined Diagnosis Related Group, in this case congestive heart failure. It also includes only admissions (OA), discharges with the same principal diagnosis and APR DRG that were not part of chains because they were not followed by readmissions.

From a retrospective standpoint, the spreadsheet data are used to identify the potential target population for the month. This population is based on the number of readmission chains and the number of only admissions combined. In order to evaluate the extent to which the target population was followed, this number is compared with the number of patients that were followed. The data in Table 3 include a target population of 25, 7 chain readmissions and 18 only admissions. This compared with 11 patients followed.

**Table 3:** Patient specific data selected potentially preventable readmissions St. Joseph's hospital health center chain month September 2011

**Account number**	**Medical record number**	**Admission date**	**Discharge date**	**Record type**	**CHF chain**	**Only admission**	**Patient followed**
1	1	09/17/11	09/19/11	IA	x		
2	1	10/18/11	10/21/11	RA	x		
3	2	09/09/11	09/13/11	IA	x		Y
4	2	09/14/11	09/19/11	RA	x		
5	3	08/31/11	09/07/11	IA	x		
6	3	09/18/11	09/23/11	RA	x		Y
7	4	09/07/11	09/10/11	IA	x		Y
8	4	09/27/11	09/30/11	RA	x		
9	4	10/22/11	10/26/11	RA	x		
10	5	08/26/11	09/09/11	IA	x		
11	5	09/11/11	09/19/11	RA	x		Y
12	6	08/27/11	09/05/11	IA	x		
13	6	09/11/11	09/21/11	RA	x		
14	7	09/12/11	09/18/11	IA	x		
15	7	09/19/11	09/22/11	RA	x		
16	8	09/07/11	09/13/11	OA		x	Y
17	9	09/11/11	09/14/11	OA		x	
18	10	09/13/11	09/15/11	OA		x	Y
19	11	08/31/11	09/15/11	OA		x	
20	12	09/11/11	09/16/11	OA		x	
21	13	09/14/11	09/19/11	OA		x	Y
22	14	09/17/11	09/20/11	OA		x	
23	15	09/12/11	09/20/11	OA		x	
24	16	09/15/11	09/21/11	OA		x	Y
25	17	09/18/11	09/21/11	OA		x	
26	18	09/17/11	09/21/11	OA		x	Y
27	19	09/22/11	09/25/11	OA		x	
28	20	09/18/11	09/26/11	OA		x	
29	21	09/06/11	09/07/11	OA		x	Y
30	22	09/02/11	09/08/11	OA		x	
31	23	09/08/11	09/09/11	OA		x	
32	24	09/18/11	09/28/11	OA		x	Y
33	25	09/25/11	09/28/11	OA		x	

The spreadsheet data also contains information that can be used to evaluate the effectiveness of interventions to reduce readmissions. This information relates to actual counts of readmissions in Table 3, rather than to the risk analysis in Table 2. For the example in Table 3, the spreadsheet identified 7 chain readmissions, based on the number of initial admissions. Within this group, 3 chain readmissions were followed by the program either as an initial admission or a readmission. The spreadsheet data suggests that, despite the program interventions, these patients were still readmitted to a hospital.

The spreadsheet provides information concerning the impact of the program for further analysis. In some cases, this analysis could indicate that program interventions were not completed or were ineffective. In others, such as patients with Major or Extreme severity of illness, it could indicate the type of clinical interventions provided in the program could not have prevented the readmission.

## Discussion

Recent efforts at health care cost containment have generated new interest in reducing hospital readmissions and other adverse outcomes as a means of improving care and saving expenses. In the case of readmissions, this is no easy task. In the hospitals of Syracuse, New York, the Potentially Preventable Readmissions software and the tools discussed in this study have provided assistance in addressing it.

One issue not discussed in this study was the preventability of hospital readmissions. Because readmission is a complex indicator, not all rehospitalizations are preventable. The degree to which a readmission is preventable relates to the severity of illness, the quality of care at all levels, continuity of care, coordination and logistics, and availability of resources. In this context, the preventability of a readmission will depend on the condition of the patient, as well as the resources available to support that patient in a specific community.

The study demonstrated that definitions of readmissions can provide the basis for management of these outcomes. In this study, the chain definition focused on characteristics of patients first and rehospitalizations secondarily, while the readmission definition focused on characteristics of individual rehospitalizations. One of the Syracuse hospitals is tracking readmissions with both definitions to benefit from both perspectives.

The study confirmed suggestions in the literature that estimating the risk of readmissions is a difficult undertaking. Both the literature and the study confirmed that it is possible to identify subindicators with higher probabilities of readmissions such as specific age levels, discharge statuses, or severity of illness categories. Using the administrative data employed in the study it was not possible to identify a single combination of indicators that could predict, with certainty, whether a patient would be readmitted. Instead, the data suggested that identifying subindicators with the highest frequencies for individual hospitals, such as elderly patients, discharges home or to long term care, and high or low severity of illness were useful in identifying and tracking patients who were readmitted.

Within this context, the study generated several finding for different patient subgroups that could be used to design followup studies. For example, studies could address whether older patients, or patients not receiving long term care, or those with higher severity of illness, are more or less likely to be readmitted to a hospital. Studies might address why the incidence of readmissions varies within these categories among different hospitals. These followup studies, possibly using randomized controlled designs, could be very useful in evaluating interventions to maximize the impact of limited clinical resources.

In addition, the breakdown into risk categories identified in this study could also be used in relation to specificx treatments and care interventions designed to improve patient outcomes. These treatments could address health status measures, daily living functions, and quality of life. If these measures are not included in widely available administrative data bases, they could be abstracted and developed by individual hospitals.

The study also demonstrated that patient specific spreadsheets were useful in evaluating patient populations tracked and the impact of readmission interventions. At the patient specific level, these spreadsheets provide more information for processes related to these outcomes than summary tables including large amounts of aggregate data.

Limitations of this study involved its design as a preliminary effort to evaluate a series of quantitative tools for addressing hospital readmissions. Another limitation involved the application of these tools within hospitals located in a single, small metropolitan area. The study was based on two specific definitions of hospital readmissions that are being used in the United States. The data concerning the risk of readmission identified in the study were based on a retrospective analysis, rather than predictive estimates.

The study suggested that each of these quantitative tools could be adapted to different hospitals and interventions addressing inpatient readmissions. Further research should identify the impact of these approaches, as well as other tools and mechanisms.

### Availability of supporting data

The data sets supporting the results of this article are available in the Hospital Executive Council repository. Because this information includes unique patient identifiers, permission from the Hospital Executive Council is required to gain access.

## Competing interests

The authors declare that they have no competing interests.

## Authors’ contributions

Each of the authors have made substantive intellectual contributions to this study. RL coordinated the acquisition and analysis of data between the hospitals and was involved in drafting the manuscript. DN was involved in the analysis and acquisition of data from Crouse Hospital, as well as drafting the manuscript. ML was involved in the analysis and acquisition of data from St. Joseph’s Hospital Health Center, as well as drafting the manuscript. Each of the authors gave their approval of the manuscript submitted. All authors read and approved the final manuscript.
